# Supplementary material for: Fcα Receptor-1-Activated Monocytes Promote B Lymphocyte Migration and IgA Isotype Switching
Source: Int J Mol Sci. 2022 Sep 22;23(19):11132. doi: 10.3390/ijms231911132 (PMC9569671; doi:10.3390/ijms231911132)
Supplement: Supplementary file 1 [file ijms-23-11132-s001.zip › ijms-1882004-supplementary.pdf]

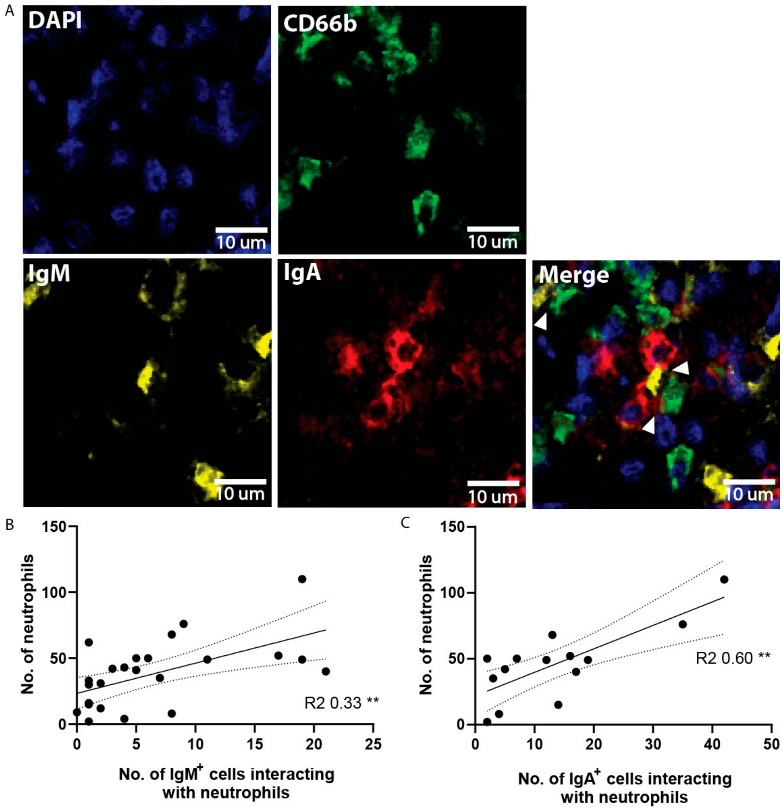

Supplemental Figure S1: Neutrophils interact with IgM⁺ and IgA⁺ B cells in inflamed colon tissue of inflammatory bowel disease patients. (A) Tissue was collected from inflamed pathological regions of the colon of inflammatory bowel disease (IBD) patients. Visualization for DNA (DAPI, blue), neutrophils (CD66b, green), IgM^+ B cells (yellow) and IgA^+ B cells (red) in inflamed colon biopsy of Crohn's disease patient. Visualization of interaction between IgM^+ or IgA^+ B lymphocytes with neutrophils is represented with white arrows. A total of 6 inflamed biopsies were assessed. Correlation between the amount of neutrophils versus the number of IgM^+ B cells (B) and IgA^+ B cells (C) interacting with at least one neutrophil within an acquired microscopy field (each dot represents one acquired field). Simple linear regression $^{**}p < 0.01$.

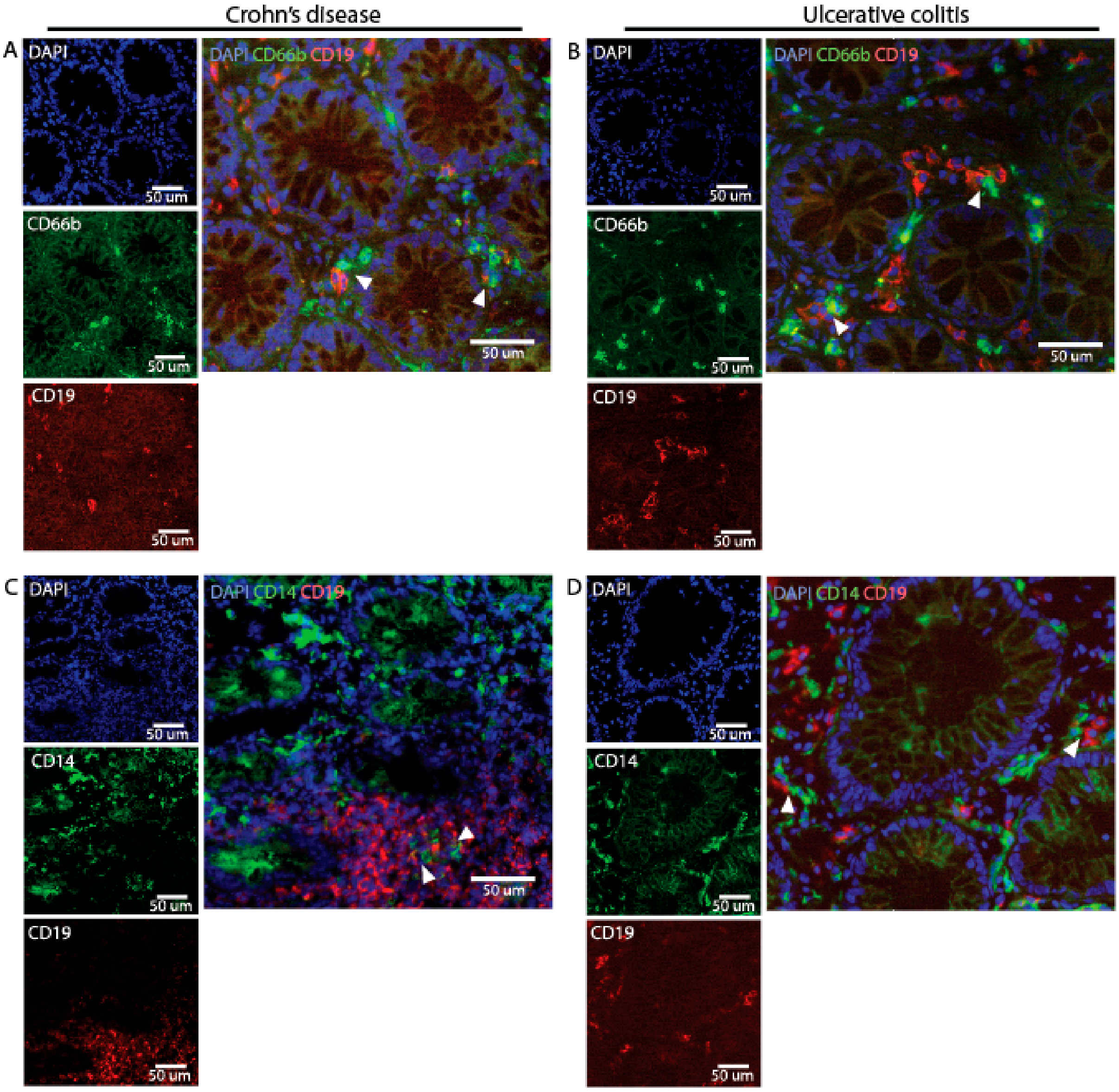

Supplemental Figure S2: Myeloid cells interact with B lymphocytes in non-inflamed colon tissue of inflammatory bowel disease patients. Tissue was collected from macroscopic non-pathological part of the colon of inflammatory bowel disease (IBD) patients. (A+B) Single staining of DNA, CD66b^{+} neutrophils and CD19^{+} cells of (A) Crohn's disease (representative sample out of n=3) and (B) Ulcerative colitis (representative sample out of n=4). Merged channels visualize the interaction (white arrows) between CD66b^{+} cells and CD19^{+} B lymphocytes. (C+D) Visualization for DNA, CD14^{+} monocytes (green) and CD19^{+} B lymphocytes in inflamed colon biopsy of patients with (C) Crohn's disease (n=4) or (D) Ulcerative colitis (n=4). Merged channels are demonstrated to visualize the interaction (white arrows) between CD14^{+} cells and CD19^{+} B lymphocytes.

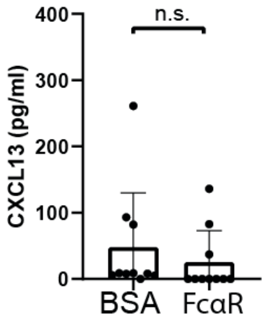

Supplemental Figure S3: Peripheral monocytes were stimulated with BSA- or IgA-coated (activating FcαRI) beads for 24 hours. Luminex was performed on supernatant of stimulated monocytes to determine C-X-C motif chemokine ligand 13 (CXCL13). Not detected protein is represented as zero. Data are presented as mean ± SD. N.S.= not significant. Student's t test; N.S $p > 0.05$.

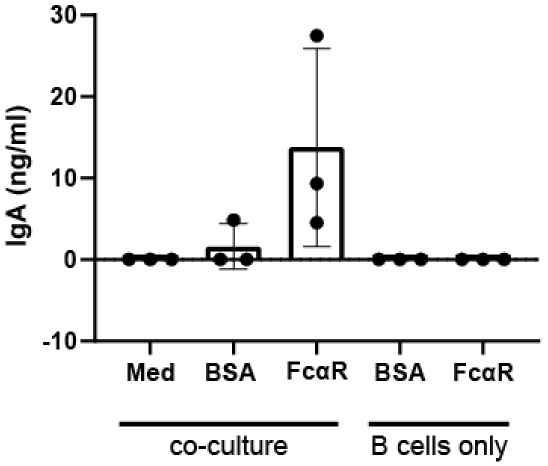

Supplemental Figure S4: Detected IgA in B cell supernatant is not caused by the IgA-coated beads. Monocytes were isolated from human blood and stimulated with BSA- or IgA-coated (activating FcαRI) beads for 30 minutes. After washing away the beads, monocytes were co-cultured with peripheral naïve B lymphocytes in the presence of T cell-dependent stimuli, including interleukin-4, anti-CD40 and anti-IgM antibodies for 7 days. Co-cultures of unstimulated monocytes were taken along as a negative control (MED). IgA secretion in supernatants are depicted (n=3).

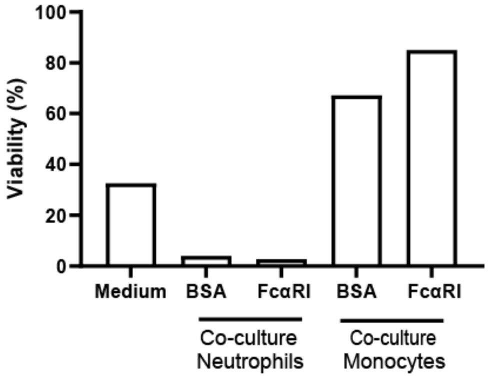

Supplemental Figure S5: Co-culture of myeloid cells affect B cell viability. Neutrophils and monocytes were isolated from human blood and stimulated with IgA-coated beads or BSA-coated beads as a negative control for 30 minutes. After washing away the beads, myeloid cells were put in co-culture with peripheral naïve B lymphocytes in the presence of T cell-dependent stimuli, including interleukin-4, anti-CD40 and anti-IgM antibodies. Representative overview of cell viability of B lymphocytes, excluding myeloid cells, after 7 days of co-culture with neutrophils (left panel) or monocytes (right panel). Naïve B cells that were cultured in medium in the absence of myeloid cells indicate baseline survival.

Supplementary Table S1: Genes of interest after RNA-sequencing analysis.

Gene	Mean BSA	Mean IgA	Mean log BSA	Mean log IgA	lfdr_groupsBSAiga	Note
IL2	Undetected	Undetected	0	0	0	
IFNA	Undetected	Undetected	0	0	0	IFNα
TNFAIP3	9248.98	30692.13	3.97	4.49	0.000	
IL6	47.09	15796.96	1.67	4.20	0.001	
TNFAIP8	2724.63	5922.12	3.44	3.77	0.001	
IL7	12.96	255.09	1.11	2.41	0.002	
CCL20	92.48	6809.07	1.97	3.83	0.003	MIP3A
IL10	113.22	570.44	2.05	2.76	0.014	
IL27	16.92	53.65	1.23	1.73	0.02	
IL12A	0.59	4.06	-0.23	0.61	0.081	
IL15	46.75	124.45	1.67	2.09	0.084	
CCL28	4.95	0.23	0.70	-0.63	0.192	
IL12B	1.91	164.92	0.28	2.22	0.258	
IFNB1	0.78	3.46	-0.11	0.54	0.298	IFN-ß
IL4	Undetected	0.04	0	-1.39794	0.451	
CD40LG	0.36	1.53	-0.44	0.19	0.472	CD40 ligan
IFNG	0.66	3.48	-0.18	0.54	0.497	IFNγ
IL13	Undetected	0.04	0.00	-1.40	0.503	
IL21	Undetected	Undetected	0	0	0.51	
CXCL12	0.95	0.57	-0.02	-0.24	0.535	
CCL19	0.42	1.24	-0.38	0.09	0.541	
TNFSF13B	212.97	284.81	2.33	2.45	0.554	BAFF
CXCL13	0.93	0.92	-0.03	-0.03	0.609	
TNFSF13	66.16	54.36	1.82	1.74	0.654	APRIL
